# Relationship between early-life stress and trait mindfulness in adulthood: a correlational study

**DOI:** 10.1186/s40359-022-01029-7

**Published:** 2023-01-20

**Authors:** Vinícius Santos de Moraes, Mariana Fernandes, Maria Neyrian de Fátima Fernandes, Larissa Bessani Hidalgo Gimenez, Elton Brás Camargo Júnior, Edilaine Cristina da Silva Gherardi-Donato

**Affiliations:** 1 Graduate Program Psychiatric Nursing at the College of Nursing, University of São Paulo at Ribeirão Preto, Ribeirão Preto, Brazil; 2 Nursing Department of the Universidade Federal do Maranhão, Av. da Universidade, S/N, Dom Afonso Felipe Gregory, Imperatriz, MA CEP: 65915-240 Brazil; 3 Nursing College, Universidade de Rio Verde, Rio Verde, GO Brazil

**Keywords:** Early stress, Stress, Mindfulness, Emotional regulation, Social support

## Abstract

**Background:**

To investigate the relationship between early-life stress (ELS) and the trait mindfulness level in workers.

**Method:**

This study is quantitative cross-sectional and correlational research with a sample of 929 workers from a Brazilian public university. ELS and mindfulness assessment was performed using the Childhood Trauma Questionnaire (CTQ) and the Five-Facet Mindfulness Questionnaire-BR (FFMQ-BR), respectively. The data were submitted to correlation tests adopting a significance level of .05 and a multivariate linear regression analysis.

**Results:**

50.0% of the participants obtained a score indicative of ELS exposure in at least one subtype among the five proposed by the CTQ, with emotional neglect predominating (63.0%). The group not exposed to emotional abuse had higher scores in the “describe—positive formulation” and “non-reactivity to inner experience” facets. Those that scored for physical abuse had higher values in “acting with awareness—autopilot”. However, the group exposed to sexual abuse obtained the highest score in the “acting with awareness—autopilot” and “acting with awareness—distraction” facets. The correlation between FFMQ-BR and CTQ overall scores showed a weak correlation with statistical significance. The multiple linear revealed that the facets of mindfulness were significantly associated by at least one type of early stress; however, no significant association was found between CTQ and FFMQ-BR overall results.

**Conclusion:**

The results showed that emotional regulation might have effectively occurred in this specific population, even with the presence of some childhood trauma.

## Introduction

It has been shown that early life stress (ELS), including physical, sexual, and emotional abuses and neglect experienced by developing children are linked to a host of physical and psychological sequelae into adulthood [1]. Among the many adverse experiences a child might encounter, ELS encompasses exposure to toxins, nutritional restriction, abuse, neglect, and limited family resources. An extensive range of cognitive, emotional, and behavioral processes are negatively impacted by chronic and severe exposure to these types of situations [2]

Developing appropriate, flexible, and adaptable responses to the demands of adult life are components of emotional regulation [3]. Emotion regulation begins with recognizing a stimulus and then establishing a meaning [4]. During the process of synthesis of emotions, both previous experiences and the behavior itself are taken into account. In accordance with existing literature, ELS negatively impacts the development of brain structures that are responsible for emotion regulation. This increases a person's vulnerability to mental and physical health disorders later in life [2]. In short, adults with an ELS history tend to have persistent hypersensitivity in the brain structure highly implicated in memory and learning of emotional content [5–7].

ELS, such as those associated with an adverse living environment narrowed in support and opportunity, are associated with harmful health outcomes [8, 9]. For example, low socioeconomic status seems to be associated with delayed cognitive development in children, including working memory, inhibitory control, cognitive flexibility, and callous behaviors [8, 9]. These lead to the transition to adulthood consequences, and ELS relates to both cognitive and associated neurobiological development [8]. These characteristics may affect mindfulness trait during lifespan.

Based on empirical studies, mindfulness is associated with the use of adaptive emotion regulation strategies that support healthy psychic functioning [10]. As a result of the growing use of mindfulness-based interventions in various contexts, research has shown that these interventions have positive effects on the physical and mental health of practitioners as well as on the brain components involved in traumatic or chronic stress [11]. Despite this, there is still a lack of research on this association.

Mindfulness as a trait is the awareness that emerges through deliberate attention in the present moment, with intention, without judgment, making the most of the current experience. All people have an innate ability to access mindfulness, portrayed in studies as mindfulness trait. This ability may be greater or less in some people, and these causes are being investigated [12].

It has been reported that trait mindfulness increases with age [13] because older age may be associated with focusing more on feelings and maximizing positive experiences. It has also been demonstrated that mindfulness meditation practice may lead to neuroplastic changes in the structure and function of brain regions associated with the regulation of attention, emotion and self-awareness increasing trait mindfulness [14]. In terms of gender, women scored higher than men on the observing subscale. In contrast, men scored higher than women on the acting with awareness subscale of the Five Factor Mindfulness Questionnaire (FFMQ) [15].

An increased level of mindfulness has been shown to be protective, with negative correlations between stress and well-being [16], and self-reported mindfulness levels are associated with a higher level of experience differentiation, reflecting on effective emotion regulation, the ability to manage and respond effectively to emotional experiences. [17]. In regard to research investigating mindfulness trait levels, we found a study which investigated and demonstrated a clear correlation between mindfulness trait, perceived stress, and well-being in healthcare professionals. The correlation between mindfulness and perceived stress was strong, while the correlation between mindfulness and well-being was medium [16].

So far, there is a gap in the literature investigating the trait mindfulness and ELS, and little is known whether this trait can be associated with the adverse effects arising from exposure to ELS. A study in the United States with a population varying between 2 and 17 years old investigated the associations between protective factors based on mindfulness and emotional, behavioral, and adversities in childhood. The results showed that mindfulness strategies contribute to consolidating new coping methods such as child resilience and stress management by parents [11]. A group-based mindfulness intervention for adolescents with ELS also demonstrated efficacy on a symptom level and potential biological changes as well [18]. According to a study involving 629 university students, resilience can mediate the effects of childhood trauma on negative emotional symptoms, while mindfulness can have a significant impact on the indirect effects of childhood trauma through resilience [19].

Considering that there is an association between the adverse effects arising from exposure to ELS and the trait mindfulness, we investigated the hypothesis that there is a negative relationship between ELS exposure and the levels of trait mindfulness. Additionally, we expected that higher trait mindfulness levels are associated with higher age and meditation practice. Therefore, this study aims to investigate the relationship between ELS and the levels of trait mindfulness in workers from a Brazilian public university.

## Methods

### Participants and procedure

This is a quantitative cross-sectional study with correlational design carried out at a Public University located in the interior of the State of São Paulo, Brazil, from July to December 2017.

The study was approved by the university’s administrative units and by the Research Ethics Committee (CAAE: 58376016.0.0000.5393). The researchers’ team approached the participants at their workplaces and invited them to participate in the study. After obtaining informed consent, we set a date for returning the completed questionnaires and communicating with researchers in case of doubt. Nine hundred twenty-nine volunteers returned the completed data collection material, corresponding to 54.51% of the total campus non-teaching staff population. We included in this study the Administrative and Technical Staff of the University, aged 18 years or older. We excluded those who worked for less than one year or answered less than 80% of the questionnaires.

### Measures

We used three questionnaires to measure the variables of interest: the sociodemographic characteristics of work and health questionnaires, the Childhood Trauma Questionnaire (CTQ) [20], and the Five-Facet Mindfulness Questionnaire (FFMQ-BR) [21].

The sociodemographic questionnaire included the following variables: age, gender, years of formal study, living with a partner, children, religious belief, meditation practice in the last 12 months, work unit, position held on campus, weekly workload, length of service in the status, has more than one employment relationship, practices some physical activity, smoking habits, alcohol consumption and use of some psychotropic medication.

The FFMQ created by Baer et al. [22] is generally widely used in psychological research, translated, and validated for Brazil as FFMQ-BR with seven facets [21]. Two facets of the original version were divided into other two facets: (1) “Describe,” which was divided into one factor containing items with positive formulation (fourth factor) and one containing the items with negative formulation (fifth factor), and (2) “Act with Awareness,” which was divided into a factor with items relating to act on autopilot (second factor) and one containing items related to act distractedly (seventh factor) [21].

The questionnaire aims to multidimensionally measure mindfulness levels: (1) Observe: notice internal and external experiences, such as sensations, emotions and thoughts; (2) Describe: label experiences in words—Subdivision: positive formulation—ease/ability to describe internal experiences through words; and negative formulation—difficulty/inability to describe inner experiences using words; (3) Acting with full attention/awareness: be focused moment by moment on the activity, instead of acting mechanically—Subdivision: autopilot—act automatically, without being aware, however focusing on the action; and distraction—act in a distracted manner without being aware, using vigilant attention, but without any specific focus on the activity; (4) Non-reactivity to the inner experience: allowing the free flow of thoughts and emotions, without being caught by them or without rejecting them; (5) Non-judgement of the inner experience: adopt a non-evaluative posture in relation to thoughts and emotions [22].

The FFMQ can measure attention levels in a wide range of populations with or without meditation experience. It is a self-administered questionnaire with 39 items scored on a Likert-type scale, and participants rated the items on a five-point Likert scale (1 = never or very rarely true through 5 = very often or always true). The maximum score of the FFMQ-BR is 195 points through the sum of each facet; and a minimum of 39 points, indicating the maximum and minimum levels of mindfulness, respectively. Higher scores indicate higher levels of mindfulness in terms of the scored facets [22].

The internal consistency of the FFMQ-BR and each facet were evaluated by Cronbach's alpha (α) and McDonald's omega (ω). The FFMQ-BR scale presented satisfactory internal consistency indices, as shown: FFMQ-BR (α = 0.814; ω = 0.825); not judging the internal experience (α = 0.768; ω = 0.772); act with conscience-autopilot (α = 0.848; ω = 0.850); observe (α = 0.778; ω = 0.781); describe—positive formulation (α = 0.838; ω = 0.839); describe—negative formulation (α = 0.720; ω = 0.722); non- reacting to external experience (α = 0.667; ω = 0.681); act with conscience-autopilot (α = 0.743; ω = 0.752).

The ELS variable was measured using the version of the CTQ with 28 items. We used the version translated, adapted, and validated to Brazilian culture [23]. Each subscale item is rated on a 5-point Likert-type scale (1 = never true through 5 = very often true). We analyzed the childhood trauma data on subscales scores and a comprehensive test of childhood trauma, with all the subscales, added together. It contains five subscales: (1) Emotional Abuse (EA) is verbal aggressions directed at the child in the sense of value or well-being or any humiliating behavior directed by an adult or older person; (2) Physical Abuse (PA) is physical aggressions suffered by a child committed by an adult or older person, representing a risk or resulting in injury; and (3) Sexual Abuse (SA) is sexual conduct between a child under 18 and an adult or older person; (4) Physical neglect (PN) is the failure of caregivers to provide the child with basic physical needs such as food, shelter, clothing, safety, and health, and (5) Emotional neglect (EN) is the failure of caregivers to meet children’s basic emotional and psychological needs, including love, care and support. Statements 10, 16, and 22 correspond to the minimization/denial control scale of the abuse experience [20].

The CTQ score is summed through points referring to each dimension's statement, totaling five scores at the end. It is necessary to score in moderate-severe or severe-extreme classifications in at least one of the subtypes for the individual with ELS. A higher score on a subscale indicates more severe childhood trauma. CTQ classifications are according to the suggested cut-off points [24]. We classified both subscale scores as well as CTQ total score into severity quintiles: “none/minimal” (EA ≤ 8, PA ≤ 7, SA = 5, EN ≤ 9, PN ≤ 7, CTQ ≤ 36), “low to moderate” (EA > 8 and ≤ 12, PA > 7 and ≤ 9, SA > 5 and ≤ 7, EN > 9 & ≤ 14, PN > 7 and ≤ 9, CTQ > 36 and ≤ 51), “moderate to severe” (EA > 12 and ≤ 15, PA > 9 and ≤ 12, SA > 7 and ≤ 12, EN > 15 and ≤ 17, PN > 9 and ≤ 12, CTQ > 51 and ≤ 68), and “severe to extreme” (EA ≥ 16, PA ≥ 13, SA ≥ 13, EN ≥ 18, PN ≥ 13, CTQ ≥ 69).

The CTQ and its subscales showed satisfactory levels of internal consistency, as described: CTQ (α = 0.888; ω = 0.904); EA (α = 0.840; ω = 0.847); PA (α = 0.752; ω = 0.790); SA (α = 0.909; ω = 0.914); EN (α = 0.838; ω = 0.844); PN (α = 0.442; ω = 0.495).

### Data analyses

The data from the FFMQ-BR instrument were presented as means, medians, and standard deviations. Using Spearman's correlation test with a significance level of *p* < .05 and *p* < .01. The correlation between the two variable varies between − 1 and + 1. Zero means there is no correlation, where 1 means a complete or perfect correlation. The strength of the correlation increases both from 0 to + 1, and 0 to − 1 [23]. Values between 0 and 0.3 (0 and − 0.3) indicate a weak positive (negative) linear relationship, 0.3 and 0.7 (− 0.3 and − 0.7) a moderate positive (negative) linear relationship, and 0.7 and 1.0 (− 0.7 and − 1.0) indicate a strong positive (negative) linear relationship. The CTQ instrument score was presented in a dichotomous form in each subtype: emotional, physical, and sexual abuse, in addition to emotional and physical neglect.

The data collected were coded and tabulated in a Microsoft Excel 2010 data sheet with double-entry typing to process the responses. Then, the database was validated using the Statistical Package for the Social Sciences® (SPSS).

Descriptive statistics of both groups' characteristics and the variables studied were performed through frequency distribution, absolute and percentage numbers, average, minimum and maximum. We used descriptive statistics for the results of the instruments according to the scores recommended in the literature. We used Spearman’s correlation test and comparison of results using the Mann–Whitney and Kruskal–Wallis non-parametric statistical tests at the significance levels of *p* < 0.05 and *p* < 0.001.

Considering the literature that suggests that multiple types of child abuse and neglect may co-occur [25], and in order to test the relationships between all CTQ subscales and FFMQ-BR facets, all five types of abuse and neglect were included as predictors in a single model in addition to age, gender and meditation practice. We performed multivariate linear regression analyzes evaluating the impact of different types of early stress on the global mindfulness score and facets. Hierarchical multiple linear regression analyzes were performed adjusting for the age and gender of the participants and the report of meditation practice given the association of these variables with mindfulness [22].

The hierarchical models were built with age and gender in the first block, more proximal to the outcome, in addition, the report of meditation practice and the different subtypes of early stress were simultaneously inserted in the second block, more distal to the outcome, through the forward method which is based on input into the model based on the partial correlation of significant independent variables (*p* <0.05). The forward method allows the evaluation of the relationship of each variable in the model by verifying the change in R^2^ (ΔR^2^).

The assumption of the linear regression analyzes were evaluated through the autocorrelation of the variables inserted in the model with values of the Durbin-Watson test between 1.83 and 2.14 and the absence of multicollinearity of the predictors was evaluated by the values of Variance Inflation Factor (VIF) and Tolerance with results equal to or less than 1.22 and 0.99, respectively.

## Results

**Fig. 1:**
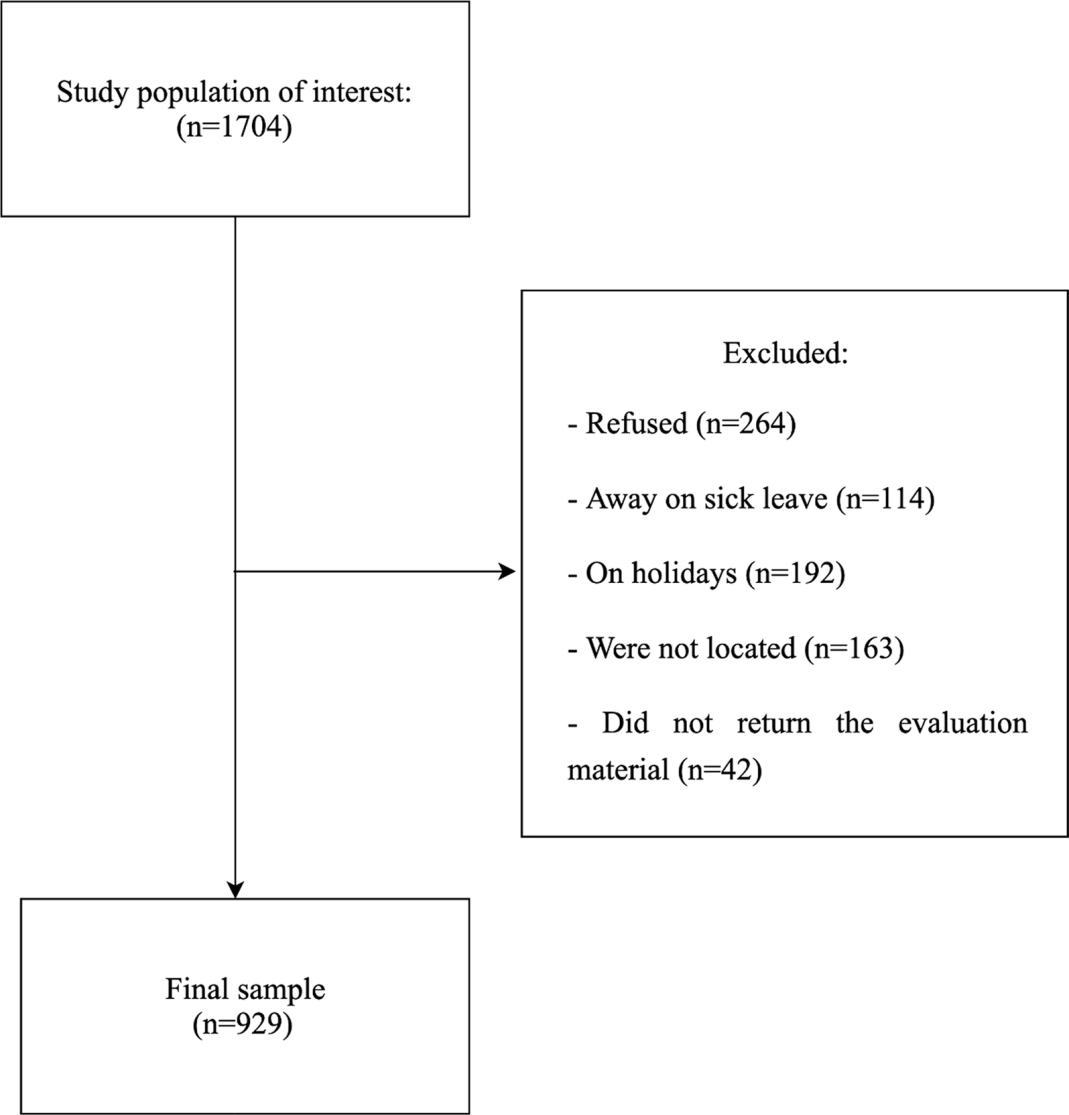
Flow diagram for study participants

The convenience sample consisted of 929 participants from the Administrative and Technical Staff of a public university in São Paulo (Brazil). This amount corresponded to 54.5% of the total of 1,704 servants in the study period. Refusals and losses represented 45.4% of the total population sample (264 refusals, 114 on sick leave; 192 were on vacation; we did not locate 163, and 42 did not return the questionnaires) (Fig. 1).

Table 1 shows the sociodemographic and health characteristics of the study population. The overall mean age of the participants was 46.1 years (standard deviation, SD: ± 10.5). There was no significant difference in terms of gender distribution (*p* = 0.140). Most participants reported living with a partner (71.0%, *p* <0.001), having children (66.2%, *p* < 0.001), and being responsible for raising them (82.5%, *p* < 0.001). There was no difference in distribution between workers who practice and do not practice religion (50.2%, *p* = 0.870).

**Table 1:** Sociodemographic, occupational and health characteristics of the participants (n = 929)

Sample characteristics	
Age, years [mean (SD)]	46.1 (10.5)
Gender [n (%)]	
Female	487 (52.5)
Live with companion (a) [n (%)]	
Yes	660 (71.0)
No	269 (29.0)
Children [n (%)]	
Yes	615 (66.2)
No	314 (33.8)
Responsible for raising the children [n (%)]	510 (82.5)
Do physical exercise [n (%)]	
Yes	519 (55.8)
No	410 (44.2)
Practice meditation [n (%)]	
Yes	191 (20.5)
No	738 (79.5)
Smoking [n (%)]	
Yes	78 (8.4)
No	851 (91.6)
Consume alchohol [n (%)]	
Yes	453 (48.7)
No	476 (51.3)
Use of psychotropic medication [n (%)]	
Yes	135 (14.5)
No	794 (85.5)
Education level [n (%)]	
Elementary	26 (2.8)
High school	221 (23.7)
Higher education	416 (44.7)
Post-graduation	266 (28.6)
Function level [n (%)]	
Basic	124 (13.3)
Technical	652 (70.1)
Superior	153 (16.4)
Relationship between work level and educational level [n(%)]	
Below	605 (65.1)
Corresponding	313 (33.6)
Above	11 (1.1)
Working time, years (SD)	18.5 (11.0)
Have another job [n (%)]	63 (6.8)
Religious practice [n (%)]	467 (50.2)

*SD* Standard deviation

Regarding health practices and conditions, 55.8% performed some physical activity (*p* < 0.001), while 20.5% practiced some meditation regularly (*p* < 0.001). We found tobacco use and alcoholic beverage consumption in 8.4% of the participants (*p* < 0.001), and 48.7% reported consuming alcohol at least twice a week. The affirmative answer for using some psychotropic medication corresponded to 14.5% (*p* < 0.001).

Also, Table 1 shows the occupational and educational characteristics of workers, that the correspondence analysis between education and the position held, 65.1% of workers occupied positions below their educational levels; 33.6% occupied compatible positions, and 1.1% occupied positions above their educational levels (*p* < 0.001). On education levels, 2.8% completed elementary school, 23.7% high school, 44.7% higher education and 28.6% post-graduation. Among the individuals in the present sample, 70.0% belonged to the technical class level (*p* < 0.001). The participants’ average employment time corresponded to 18.5 years (SD = 11.0), and only 6.8% of the participants reported having a second job (*p* < 0.001).

**Fig. 2:**
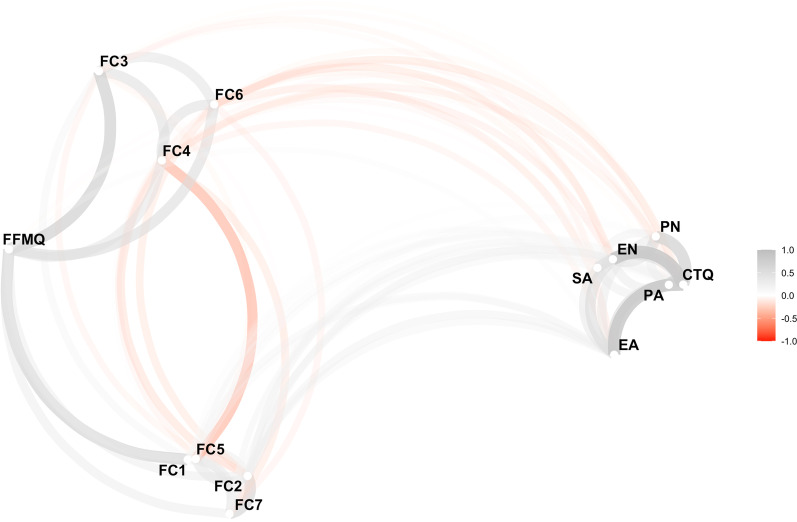
Spearman correlation between Early-Life Stress (CTQ) and Mindfulness facets (FFMQ-BR) of participants (n = 929). CTQ, Childhood Trauma Questionnaire; EA, Emotional abuse; PA, Physical abuse; SA, Sexual abuse; PN, Physical neglect; EN, Emotional neglect; FFMQ-BR, Five Facet Mindfulness Questionnaire-Brazilian; FC, Facet; Facet 1, Non-judgement of the inner experience; Facet 2, Acting with awareness—autopilot; Facet 3, Observe; Facet 4, Describe—positive formulation; Facet 5, Describe—negative formulation; Facet 6, Non-reactivity to inner experience; Facet 7, Acting with awareness—distraction; ρ, Spearman’s correlation = − 1 to + 1, *p < 0.05, **p < 0.01. The proximity of variables is determined using multidimensional clustering. Variable that are highly correlated are clustered together. Gray paths indicate positive correlations, and red paths are negative correlations. The width and transparency of the path represent the strength of the correlation (wider and less transparent = stronger correlation)

Figure 2 shows a correlation between each CTQ subtype and the mindfulness level (FFMQ) through individualized facets. The Spearman’s Correlation test was used with a value of *p* < 0.05 and *p* < 0.001.

In general, the total results of FFMQ-BR and CTQ showed a weak correlation with statistical significance (r = 0.036, *p* = 0.03). However, the FFMQ-BR facet “non-reactivity to inner experience”, showed weak correlation with statistically significant values, namely: EA (r = 0.206, *p* < 0.001); EN (r = 0.119, *p* < 0.001) and PA (r = 0.108, *p* < 0.001). The values for the “acting with awareness—autopilot” facet showed moderated correlation with statistical relevance in EA (r = 0.312, *p* < 0.001), and weak correlation with EN (r = 0.274, *p* < 0.001), SA (r = 0.144, *p* < 0.001), PN (r = 0.112, *p* < 0.001), and PA (r = 0.086, *p* < 0.001).

There was a weak, negative correlation between “observe” facet and PN (r =  − 0.126) with significant relationship (*p* < 0.001). The “describe—positive formulation” facet was negative, weak correlated with significant relationship with EN (r =  − 0.186, *p* < 0.001), EA (r =  − 0.160, *p* < 0.001), PN (r =  − 0.078, *p* < 0.01), and SA (r =  − 0.075, *p* < 0.02). The facet “describe negative formulation” showed a weak correlation with EA (r = 0.174, *p* < 0.001), EN (r = 0.139, *p* < 0.01), PN (r = 0.099, *p* < 0.001), SA (r = 0.077, *p* < 0.01), and PA (r = 0.072, *p* < 0.03).

Finally, in relation to the “acting with awareness-distraction” facet there was a weak correlation with the EA (r = 0.236, *p* < 0.001), EN (r = 0.185, *p* < 0.001), SA (r = 0.109, *p* < 0.001), PN (r = 0.079, *p* < 0.01), and PA (r = 0.071, *p* < 0.03) subtypes.

We performed multiple linear regression analyzes to verify how the models structured through the assessment of age, gender and history of meditation practice, and types of ELS are associated with the levels of different facets and the total mindfulness score (Table 2).

**Table 2:** Multivariate linear regression coefficients of early stress types as predictors of mindfulness levels

Predictor	B	Std. Error	Beta	t	*p*	R^2^_adjusted_	*ΔR*^2^
*Facet 1*							
Constant	17.986	1.358	–	13.247	< 0.001	–	–
Age	0.038	0.020	− 0.061	− 1.872	0.062	0.004	0.005
Emotional Abuse	2.797	0.803	0.122	3.481	0.001	0.025	0.022
Emotional neglect	1.443	0.700	0.072	2.063	0.039	0.028	0.004
*Facet 2*							
Constant	9.083	0.967	–	9.393	< 0.001	–	–
Age	− 0.109	0.013	− 0.260	− 8.432	< 0.001	0.069	0.069
Gender	0.548	0.273	0.062	2.007	0.045	0.072	0.006
Emotional neglect	1.946	0.454	0.144	4.290	< 0.001	0.107	0.040
Sexual Abuse	2.225	0.583	0.122	3.819	< 0.001	0.124	0.018
Emotional Abuse	1.224	0.527	0.079	2.322	0.020	0.128	0.005
*Facet 3*							
Constant	29.218	1.653	–	17.674	< 0.001	–	–
Age	0.068	0.021	0.132	4.132	< 0.001	0.021	0.022
Meditation	− 2.817	0.544	− 0.166	− 5.179	< 0.001	0.047	0.026
Emotional neglect	− 2.385	0.669	− 0.114	− 3.565	< 0.001	0.058	0.013
*Facet 4*							
Constant	19.032	1.317	–	14.446	< 0.001	–	–
Age	0.060	0.015	0.126	3.897	< 0.001	0.019	0.020
Emotional abuse	− 1.868	0.608	− 0.107	3.897	0.002	0.036	0.018
Meditation	− 1.045	0.398	− 0.085	− 2.625	0.009	0.042	0.007
Emotional neglect	− 1.204	0.529	− 0.079	− 2.277	0.023	0.046	0.005
*Facet 5*							
Constant	4.980	0.356	–	14.009	< 0.001	–	–
Emotional abuse	1.245	0.316	0.129	3.944	< 0.001	0.015	0.017
*Facet 6*							
Constant	25.564	1.429	–	17.883	< 0.001	–	–
Age	0.075	0.016	0.154	4.831	< 0.001	0.029	0.030
Gender	− 0.861	0.328	− 0.083	− 2.627	0.009	0.037	0.009
Emotional abuse	− 2.078	0.634	− 0.114	− 3.279	0.001	0.053	0.026
Meditation	− 1.599	0.407	− 0.125	− 3.929	< 0.001	0.068	0.015
Emotional neglect	− 1.345	0.545	− 0.085	− 2.470	0.014	0.075	0.008
Sexual abuse	− 1.627	0.700	− 0.077	− 2.325	0.020	0.079	0.005
*Facet 7*							
Constant	6.523	0.663	–	9.839	< 0.001	–	–
Emotional abuse	1.482	0.348	0.140	4.254	< 0.001	0.067	0.027
Sexual abuse	1.186	0.407	0.095	2.915	0.004	0.075	0.008
*FFMQ total*							
Constant	114.388	2.558	–	44.715	< 0.001	–	–
Meditation	− 4.341	1.391	− 0.102	− 3.120	0.002	0.009	0.010

FFMQ-BR, Five Facet Mindfulness Questionnaire-Brazilian. FC, Facet; Facet 1, Non-judgement of the inner experience; Facet 2, Acting with awareness—autopilot; Facet 3, Observe; Facet 4, Describe—positive formulation; Facet 5, Describe—negative formulation; Facet 6, Non-reactivity to inner experience; Facet 7, Acting with awareness—distraction

The facets of mindfulness were significantly associated by at least one type of early stress; however, no significant association was found between CTQ and FFMQ-BR overall results. Table 2 presents the coefficients for all significant predictors sequentially to the strength of the association of ELS types on mindfulness facet scores. Facet 2 was the most associated by ELS with predictors explaining 12% of the outcome [F(4,922) = 34,957; *p* < .001; adjusted R^2^ = 0.128]. As can be seen, the subtype of ELS, emotional neglect, was the most strongly associated with the facet 2 of the FFMQ-BR, explaining 4% of the outcome.

Furthermore, the different types of ELS explained 2.8% of facet 1 [F(3.923) = 10.031; adjusted R^2^ = 0.028], 5.8% of facet 3 [F(3.923) = 20.176; adjusted R^2^ = 0.058], 4.6% of facet 4 [F(4.922) = 12.150; adjusted R^2^ = 0.046], 1.6% of facet 5 [F(2.924) = 8.744; adjusted R^2^ = 0.016], 7.9% of facet 6 [F(5.921) = 16.899; Adjusted R^2^ = 0.079] and 7.5% of facet 7 [F(3.923) = 26.003; adjusted R^2^ = 0.075], these models were significant (*p* < 0.001). The results show that emotional abuse and emotional neglect were the most associated types of early stress in varying levels of mindfulness (Table 2).

## Discussion

The present study proposed investigating how ELS can associate with trait mindfulness levels in a population of workers in a public university's administrative-technical category. After analyzing the scores related to the CTQ and FFMQ-BR, participants exposed to some ELS subtypes scored higher than those who were not exposed to ELS in the mindfulness facets' general distribution.

The correlation tests between the variables in question predominantly showed values of positive coefficients, with emphasis on the “non-judgement of the inner experience”, “acting with autopilot conscience-autopilot”, “acting with awareness-distraction”, and “non-reactivity to inner experience” facets. Moreover, “emotional neglect” and the “observe” facet stand out among the findings containing negative correlations.

The correlation between childhood trauma and symptoms of psychological disorders in a similar adult population pointed out that the presence of trauma favors the appearance of anxiety, somatization, psychoticism, paranoid ideation, compulsive obsession, hostility, phobia, and depression symptoms [26].

In searching for tools to mitigate the causes of negative experiences for the individual, scientific evidence demonstrates how much mindfulness favors mental health and emotional regulation. Stress management decreased reactivity and openness to observe negative thoughts and emotions [25–28].

It is essential to note that the act of abandoning the child or adolescent by relatives or caregivers is characterized as one of the components of emotional neglect and can directly influence the development of ELS [29, 30]. Thus, the biosocial theory stands out in this context in which the vital role of the person providing care in developing (or not) trauma in childhood [31]. Social support for this individual during childhood and adolescence is one of the determining factors for developing effective coping strategies, satisfactory corroborating levels of health [28, 31].

Regarding the “non-reactivity to inner experience” facet, our results demonstrate that the participants exposed to EA and PN have higher averages than individuals not exposed to such experiences. The correlation values between these previously mentioned subtypes and the others were negative. Thus, it is possible to understand that some adverse experiences related to contexts of abuse and or neglect of a physical, emotional, or sexual character tend to result in greater reactivity towards inner experiences. Differently, a meta-analysis containing 148 studies found positive correlations of the “non-reactivity to inner experience” facet with affective symptoms in a population of nonmeditators [32].

The analysis of the “non-judgment of the inner” facet showed positive correlation values with the presence of ELS, according to the FFMQ-BR evaluation [21]. Our study indicates that the individual tends not to label their emotions and thoughts more frequently. In contrast, we observed a negative correlation in the original FFMQ questionnaire's validation study between the referred facet and functional constructs for the individual’s health, such as “openness to experience” and “emotional intelligence” [33].

Our results agree with other studies’ findings that showed negative correlation values between the same facet and deleterious psychological symptoms [33]. Thus, concerning the “non-reactivity to inner experience” and “non-judgment of the inner experience” facets of our results, we hypothesize that even though these individuals are more aware and judging less often, the fact that they are reacting with greater intensity to their inner experiences can demonstrate losses in coping strategies for situations related to ELS.

In the case of the “observe” facet, the present study's findings demonstrated a single negative correlation with EN. There was no correlation with the other subtypes. These results match those observed in studies that reveal the same outcome in a sample of nonmeditating adults [22, 32]. These findings confirm previous research, including the author's investigation of the scale in which it highlights that this facet does not access the attentional aspect of individuals without experience with meditative practices [22, 34].

Everyone, in general, seeks coping methods when faced with a stressor, which in this specific case is childhood trauma. Hence, emotion regulation is within the scope of coping strategies as a potential and innate component. The ability to promote strong and effective responses to everyday stressors defines the term emotional regulation [35]. Scientific evidence points out how adverse early experiences contribute to inadequate adaptation to these objective and pathological conditions such as depression, anxiety, and cancer [31, 32, 34].

The effects of childhood trauma on the nervous system are widely discussed in the literature. Studies suggest that children may develop dissociative or somatic symptoms throughout life because they have experienced a dissociative adaptive defense in response to trauma, or also symptoms such as anxiety, sleep disturbances, hyperactivity in face of a hyperarousal adaptation [36].

Psychological trauma affects brain development differently in girls and boys with a history of childhood trauma. In a previous study, the presence of childhood trauma and the volumes of specific brain areas revealed increased volumes for girls and decreased volumes for boys in the hippocampal and parahippocampal regions for children with a history of high-level childhood trauma—children who reported 4 or more traumas [37].

Predominantly, the facets “non-judgment of the inner” and “acting with awareness” reveal a negative correlation with affective symptoms, while moderate correlations are presented in the facet "non-reactivity to inner experience” and “describe”. In the “observe” facet, correlations with affective symptoms are not evidenced. The “non-judgment of the inner” and “acting with awareness” facets are essential to understand the relationship between trait mindfulness and negative affective symptoms, because they are components of emotional regulation [32, 38].

Our results elucidate that those individuals with a history of ELS had higher averages in these specific facets in all subtypes of the CTQ. Accordingly, there is a possibility that there may be some association with the "non-judgment of inner experience” and “acting with awareness” facets specifically for this population when scoring for the presence of some trauma.

The present findings corroborate the literature showing a predominance of EN among the participants exposed to at least one subtype (n = 46; 4.9%) of the current sample [32]. As previously mentioned, regarding EN, this component is fundamental to aggravating proactive strategies to regulate emotion [29, 30].

The participants in this study have sociodemographic characteristics of health and work within the context of social support. Having a working position within a public university, being part of the highest percentage with higher education, having stability and financial security are factors that demonstrate that these participants, in some way, received social support during childhood and adolescence. Thus, they could create a possibility to develop the necessary skills to deal with adverse experiences from the past.

The correlation tests between the types of childhood trauma (CTQ) and mindfulness facets (FFMQ-BR) in our study found a weak correlation, statistically significant in most of the variables. Trauma exposure during childhood may be associated with some aspects of trait mindfulness levels, however, there are several other relevant factors to consider.

A significant limitation to this study needs to be acknowledged. During data collection, the university changed the rector, so the population's climate of insecurity and high stress was clearly noted.

A cross-sectional design is a snapshot of a given situation, making it difficult to make causal inferences of the factors related to the mindfulness level of this population. Also, the high number of people recruited and approached in person proved to be a challenge. However, personal approaches favored individuals’ adherence since the opportunity to be close and establish direct contact with the participants seemed relevant and enlightening for those involved. It is also important to note that researchers' veracity and engagement in the data collection, covering the entire university campus, and approaching each participant in person favored the recruitment.

Another limitation was the application of the FFMQ-BR questionnaire. Despite being self-applied, the participant's understanding of the questions proved outdated, especially with those questions that started with the word “non”. Thus, it was not uncommon for researchers to assist in applying the questionnaire. The number of questions in this instrument seems to be a limiting factor since the participants became tired when answering them. However, this questionnaire’s choice is because it has been validated and translated in six countries and measures the mindfulness traits in populations with and without meditation experience [20, 35, 39].

Finally, the Cronbach’s alpha in this study was lower than 0.7. After we double checked and found no missing data or unreasonable responses, we decided to use the primary measure. We based our decision on previous studies [40, 41] in which acceptance reliability was less than 0.7.

## Conclusion

Understanding that childhood trauma is associated with trait mindfulness levels in adulthood encourages an invitation to look more carefully at children and adolescents since they can suffer significant mental health consequences. Our findings may even emphasize the importance of improving emotional regulation concerning the theme of ELS. Thus, it is necessary to create strategies aimed at the tertiary health sector and mainly at the primary and secondary sectors to favor health promotion, disease prevention, and the adequacy of public and private financial investments directed to mental health.

In summary, our results corroborate the literature, showing that childhood trauma is related to trait mindfulness levels in a population of workers in the administrative-technical category.

## Data Availability

All data generated and analysed during this study are included in this published article.
